# What motivates the preservation of endangered intangible cultural heritage? Aesthetic attraction versus cultural identity-a case study of Liaoxi Puppet Opera

**DOI:** 10.3389/fpsyg.2026.1870437

**Published:** 2026-07-22

**Authors:** Xiaoxiao Dou, Xiaona Xie, Yannan Zhang

**Affiliations:** College of Arts, Northeastern University, Shenyang, China

**Keywords:** cultural identity, flow experience, intangible cultural heritage (ICH), Liaoxi Puppet Opera, S-O-R framework, structural equation modeling

## Abstract

**Introduction:**

Against the backdrop of digital transformation, the paradigm for protecting endangered Intangible Cultural Heritage (ICH) is shifting from static preservation to living transmission. However, the psychological mechanisms converting the unique visual aesthetics of heritage into audience participation intention remain underexplored. This study focuses on the endangered Liaoxi Puppet Opera in Northern China to better understand audiences’ preferences for ICH.

**Methods:**

We constructed an integrated model based on the Stimulus-Organism-Response (S-O-R) framework. To ensure high ecological validity, the study utilized exclusive live performance recordings and primary source images from a national-level inheritor as experimental stimuli. Data from 440 respondents were analyzed using Structural Equation Modeling (SEM) and Bootstrapping.

**Results:**

The results identify a dual-path pattern of associations: (1) Initial Association: As an environmental stimulus, Puppet Perception is significantly associated with both the audience’s Flow Experience (the hedonic path) and Cultural Identity (the eudaimonic path). (2) Sequential Mechanism: A significant serial mediation path (Puppet Perception → Flow Experience → Cultural Identity → Participation Intention) was identified. (3) Dominance of Identity: The predictive strength of Cultural Identity on participation intention (β ≈ 0.48) is significantly stronger than that of Flow Experience (β ≈ 0.24).

**Discussion:**

Sensory pleasure (Flow) acts as a crucial prerequisite hook that facilitates deep cultural resonance. While aesthetic pleasure initiates the experience, awakened meaning-seeking (Cultural Identity) anchors sustainable heritage conservation. These findings add critical nuance to the entertainment-first marketing paradigm, suggesting that the key psychological predictor for maintaining the vitality of endangered ICH lies in awakening deep cultural genes through the aesthetic experience.

## Introduction

As a living witness to human cultural diversity, the Intangible Cultural Heritage (ICH) carries a certain type of historical memory and emotional genes of specific ethnic groups and serves as the link to maintain the national spiritual homeland (Kurin, 2004). However, under the double impact of globalization and modernization, the living space of traditional culture is under unprecedented pressure (Hafstein, 2008). Especially for traditional performing arts that rely on Oral transmission and practical demonstration, they not only face the death of physical form, but also face a profound crisis of social function degradation and intergenerational audience disconnect (Logan et al., 2015). Against this background, the paradigm of ICH protection is shifting from the early rescue documentation to more sustainable living transmission (Harrison, 2012). It is essential to rebuild the spiritual connection between ICH and modern people and to make it return to the real life of contemporary audiences (Smith, 2006). In China, puppetry is known as a living fossil that helps us trace the origins of regional culture. It refracts stable genes of folk traditions for thousands of years, concretizes historical allusions and folk beliefs through unique artistic programs, and deeply interprets the spiritual connotation and aesthetic pursuit of the Chinese nation (Xie and Zhang, 2024). It is said in *People’s Daily*, “Cultural heritage not only vividly describes the past, but also profoundly affects the present and the future” (Commentator TNS, 2024). As an outstanding representative of puppetry in northern China, Liaoxi Puppet Opera occupies an irreplaceable position in China’s ICH map. Geographically, “Liaoxi” refers to the region west of the Liao River in Liaoning Province (encompassing modern-day cities such as Jinzhou, Chaoyang, and Huludao), and this puppetry art form is the quintessential cultural expression of this specific area. It originated from the tide of immigration and trade in the Ming and Qing Dynasties. It is the product of a deep integration of puppet skills from all sides and regional culture in Liaoxi Corridor, a crucial geographical and cultural bridge between North and Northeast China, filling the gap in northeast puppet art. From a historical perspective, it has high academic value and revolutionary heritage: it once gave birth to the first national puppet team in New China - the Puppet Team of Liaoning Cultural and Industrial Troupe. Its core members later were assigned by central government to construct Chinese National Puppet Art Troupe, laying the foundation for the development of contemporary Chinese puppet art. The history of rises and falls of Liaoxi Puppet Opera in its development is a microcosm of the tenacious survival of Chinese folk arts in the changing times over the last hundred years. From artistic dimensions, Liaoxi Puppet Opera carries a unique regional aesthetic gene, which partially strengthens the people’s sense of geographical, cultural and social belongings, and becomes an important symbol for maintaining the cultural memory of the integration of Central Plains and Northeast China.

However, despite its extremely high historical and cultural value, the Liaoxi Puppet Opera has faced with a severe existential crisis in contemporary times. As a national ICH, it is still troubled by insufficient participation under the siege of digital media and modern entertainment (Van Dijck, 2013). On the one hand, the crisis of succession has become increasingly prominent. The appeal of traditional master-apprentice model has significantly waned in the face of market economy pressures, which results in lack of young practitioners, posing a severe risk to the preservation of core skills (Song et al., 2019). On the other hand, the loss of audiences makes matters worse. Under the impact of short videos, the younger generation lose their interests in puppetry, and the similar traditional artistic genres have gradually been replaced by foreign animation videos, resulting in the collapse of the intergenerational transmission chain of Cultural Identity (Ye, 2022). The lack of social participation has made this art form, which was once active in the rural society, gradually disappeared from people’s daily life (Ibrahim et al., 2025). This dilemma shows that it is far from enough to reverse the decline only relying on government support, and there is an urgent need to explore an endogenous primary predictor to push the audiences to actively participate in protection (Zou et al., 2025). In response to the protection of ICH, the existing literature mostly focusses on the discussion of the supply side, such as historical verification, description of artistic characteristics or policy and system design (Kuah and Liu, 2016), while from the demand side (audience perspective), there is a relative lack of empirical research on quantitative analysis of participation motivation. We still do not know how the external visual aesthetics (Stimulus) of Liaoxi puppetry, which has both visual wonder and cultural depth, is transformed into internal psychological experience (Organism), and then is associated with participation intention (Response)? Is the entertaining sensory experience more attractive to the audience, or the deep Cultural Identity is a stronger predictor of behavior? Existing research has not yet fully explored the associative patterns within this psychological black box. In order to fill this gap, this study introduces the classic theory in the field of environmental psychology-the Stimulus-Organism-Response (S-O-R) model (Mehrabian and Russell, 1974), and builds an integrated dual-path research framework. This study defines the unique Puppet Perception (such as styling and skills) of Liaoxi puppetry as the core environmental stimulus (S); Flow Experience (the hedonic path) and Cultural Identity (the eudaimonic path) as the Organism (O); Participation Intention as the final behavioral response (R). This study tries to answer a core proposition through empirical data: in the living transmission of endangered ICH, is it more important to start with aesthetic attraction or land on Cultural Identity? By doing so, this study extends the S-O-R framework by demonstrating that hedonic states (Flow) and eudaimonic evaluations (Cultural Identity) are not merely parallel outcomes but operate in a sequential associative pathway within the context of endangered heritage. Specifically, this study aims to solve the following two problems.

*RQ1*: How can the unique visual perception of Liaoxi puppetry relate to the audience’s Flow Experience and Cultural Identity through different psychological paths?

*RQ2*: Between Cultural Identity and Flow Experience, which one plays a more decisive role in predicting the audience’s participation intention?

To address these research questions, this study empirically tests the proposed model using data from 440 respondents. The conclusions indicate that visual aesthetics successfully activate both Flow Experience and Cultural Identity, with the latter serving as the dominant predictor of participation intention. In terms of theoretical innovation, this study extends the S-O-R framework by uncovering a sequential associative pathway, demonstrating that hedonic immersion (Flow) acts as a necessary facilitator for deep eudaimonic resonance (Cultural Identity). Finally, based on these findings, the paper proposes practical improvement suggestions for ICH management, advocating for a strategic shift from entertainment-first sensory displays to value-driven cultural stewardship, thereby ensuring the sustainable living transmission of endangered heritage.

## Literature review

### The S-O-R framework and its suitability in the ICH context

The S-O-R theory (Stimulus-Organism-Response) was first proposed by environmental psychologists Mehrabian and Russell (1974) (Mehrabian and Russell, 1974). In this framework, stimulus (S) represents the environmental cues that elicit individual reactions. In this context, the organism (O) represents the internal psychological state (including emotion and cognition) after the individual is stimulated, while the response (R) represents the behavioral intention that is finally shown (Jacoby, 2002). At present, the S-O-R theory is widely used in the fields of cognitive science and behavioral science, and has been explored in terms of Consumer Satisfaction, Word-of-Mouth (WOM), etc (Chen et al., 2022). ICH experience is essentially an emotional resonance process promoted by highly aesthetic stimulation, rather than purely rational decision-making (Li S. et al., 2024). Compared with the traditional model that focusses on utilitarian evaluation, the S-O-R framework can more keenly capture the dynamic transformation from sensory contact to psychological arousal. In existing research, the S-O-R theory is often used to evaluate the knowledge sharing intention of heritage digitalization (Zhang and Zhang, 2025), the consumption and purchase intention of heritage products (Zhang et al., 2023), and the importance of heritage activities (Grcheva and Oktay Vehbi, 2021). Based on this, the S-O-R framework also has significant theoretical suitability in analyzing the living inheritance mechanism of Liaoxi Puppet Operas.

Stimulus (S): Puppet Perception Quality. In the model of this study, the core stimulus source is operated as Puppet Perception Quality (PPQ), that is, the audience’s comprehensive evaluation of the unique aesthetic characteristics, artistic skills and folk narrative energy of Liaoxi Puppet Operas. As an outstanding representative of puppetry in northern China, Liaoxi Puppet art presents an artistic style belonging to the northern region, which constitutes a highly recognizable visual feature. The northeast plain is vast, and the people’s customs are simple and heroic. The Liaoxi Corridor is a transportation fortress connecting the Northeast and the Central Plains. Here, different ethnic groups and cultures blend and collide with each other. Many details in the Liaoxi Puppet Opera vividly reproduce the living customs of the northeast region. For example, the life scene of *maidens smoking long-stemmed pipes* (shiba guniang diao yandai), sartorial fashion of *wearing fur coats fur-side out* (fanchuan piao maozaiwai), the dietary habit of *gathering wild vegetables with birch-bark baskets* (shupi beikuang caishancai), and the festive activity of *participating in Shamanic ritual dances* (gensui saman tiaoshenhan).

**Figure 1:**
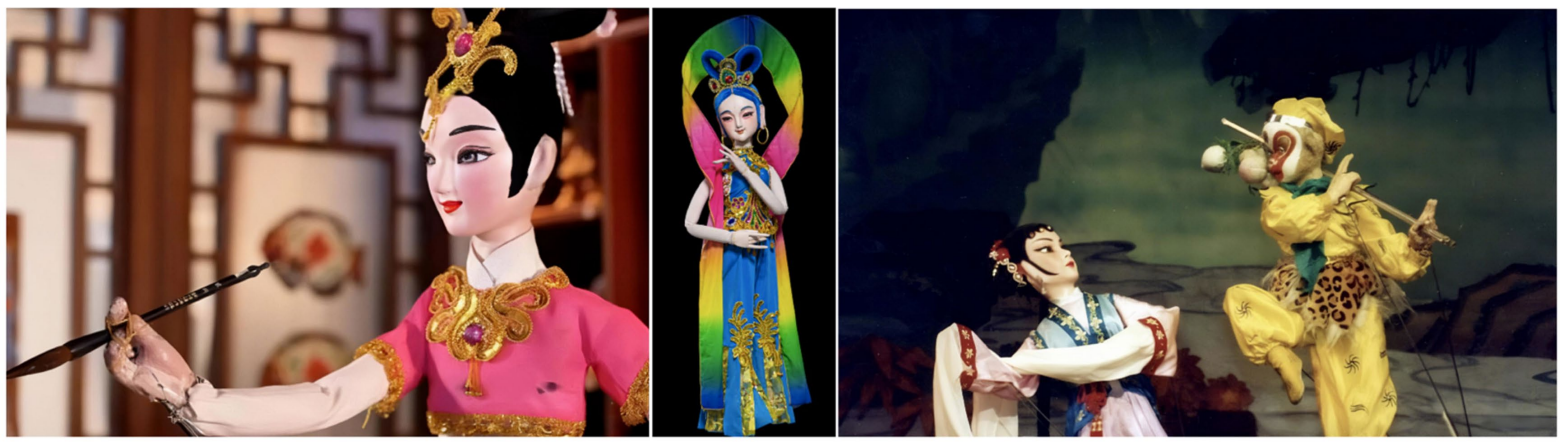
Patterns on puppet costumes and performance stills from Liaoxi.

First, in terms of plastic arts, Liaoxi puppetry adheres to the aesthetic principle of realism. Its puppet head is delicately carved, and the clothing patterns are deeply imbued with rich regional folk symbols (Xie and Zhang, 2024). These elements not only constitute sensory stimulation, but also serve as a social semiotic code, conveying the specific ethical concepts and aesthetic genes of the Liaoxi corridor area (Figure 1). Bitner once pointed out (Bitner, 1992) that visual clues in the material environment can significantly shape the internal perception of individuals. This highly realistic presentation full of folk metaphors is the first entrance to attract the attention of the audience.

**Figure 2:**
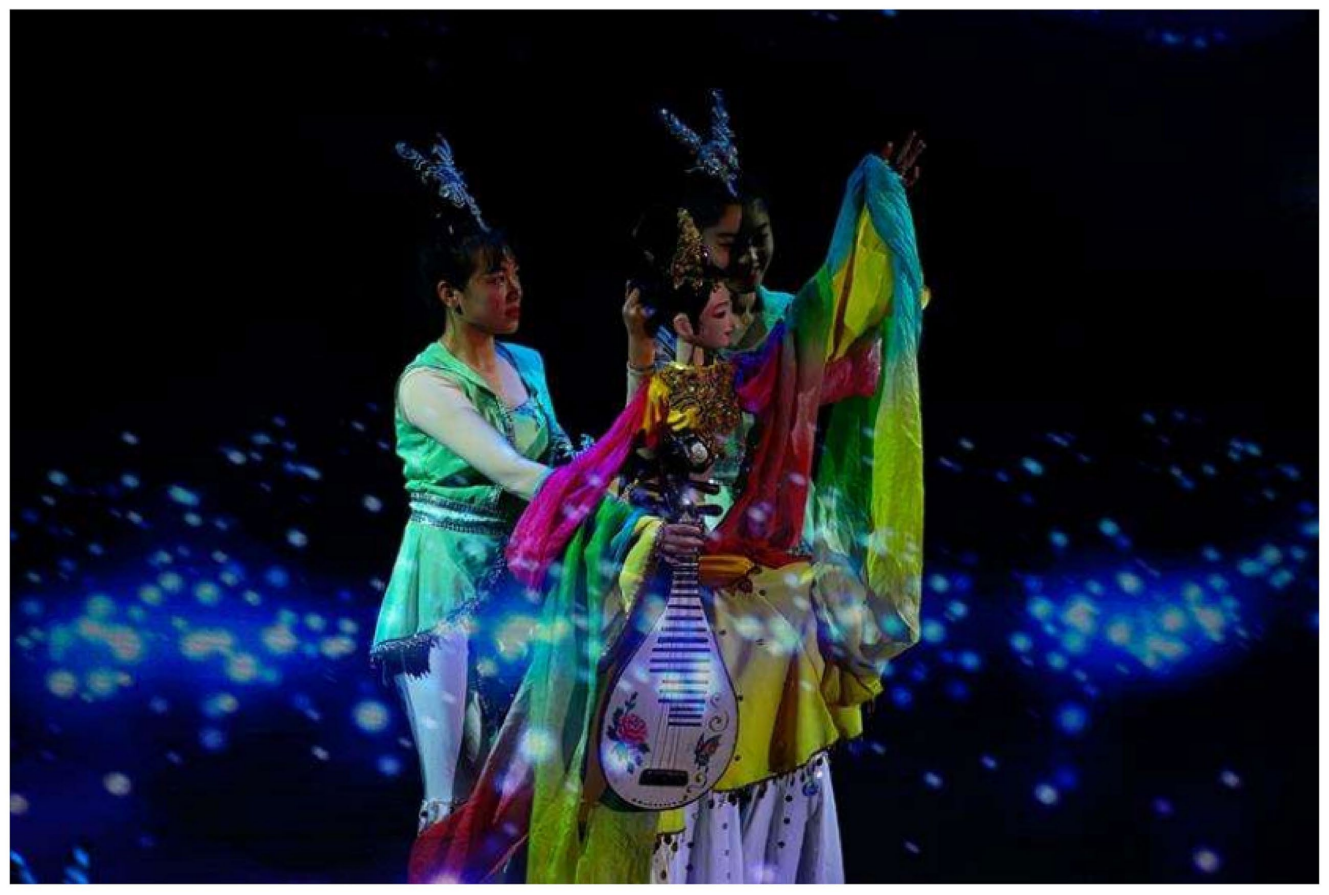
Stage photo of the Liaoxi Puppet Opera *Apsaras*, showing the integration of soft body puppets and modern stage effects.

Secondly, in terms of performance programs and repertoire, the original Pistol-grip control mechanism manipulation device gives puppets far more expressiveness than traditional Rod Puppets, enabling them to complete difficult movements such as fire-breathing and changing faces, forming a shocking visual spectacle. In the play Apsaras (Figure 2), the organic integration of Soft-bodied puppets and Modern sound, light, and electronic technologies further strengthens the impact of environmental stimulation. In addition, classic plays such as *Pigsy Carries His Wife* (zhubajie bei xifu), *Monkey King Thrice Beats the White Bone Demon* (san da baigujing), *The Story of Legs* (tui de gushi), *Little Bell, The Charm of Liaoxi Puppetry* (liaoxi ou yun), and *Face-Changing* (bian lian) have vividly portrayed the daily life scenes of the people in Liaoning, carrying the regional aesthetic Lineage and strengthening the geographical belonging of the people. Kirillova emphasized (Kirillova et al., 2014) that aesthetic characteristics are the primary factors that elicit emotional reactions. This strong stimulation, which is composed of exquisite skills and geographical identification symbols, is the material basis for inducing the transformation of the audience’s psychological Organism (O).

Organism (O): The Dual-Path Mechanism. In the S-O-R framework, the Organism is the central black box that processes external information and produces reactions. In view of the dual attributes of performing arts and cultural carriers, this study has established two unique psychological states as variables: Flow Experience and Cultural Identity. Flow Experience (The Hedonic Path: Flow is defined as an overall feeling generated when people concentrate on an activity (Csikszentmihalyi, 2000). In the context of puppetry, it refers to the state of immersion, pleasure and distorted sense of time when the audience is attracted by the visual spectacle of the performance. It represents the immediate and sensory psychological enrollment. Cultural Identity (eudaimonic path): Cultural Identity refers to the sense of group belonging based on shared cultural codes and values (Hall, 2015). In this study, it represents the audience’s deep emotional connection and value resonance awakened by the folk symbols and regional narratives embedded in puppetry. It marks the sublimation from sensory enjoyment to spiritual belonging.

Response (R): Participation Intention. The final reaction (R) in this study is manipulated into the Participation Intention. According to the theory of planned behavior (Ajzen, 1991), the willingness to act is the most direct predictor of actual behavior. For endangered ICH, participation is not limited to passive viewing but also includes a wider range of supportive behavior. Therefore, this study defines the Participation Intention as the subjective will of the audience to engage in protective actions, including re-viewing, recommending to others and actively learning relevant heritage knowledge (Xie J. et al., 2024).

**Figure 3:**
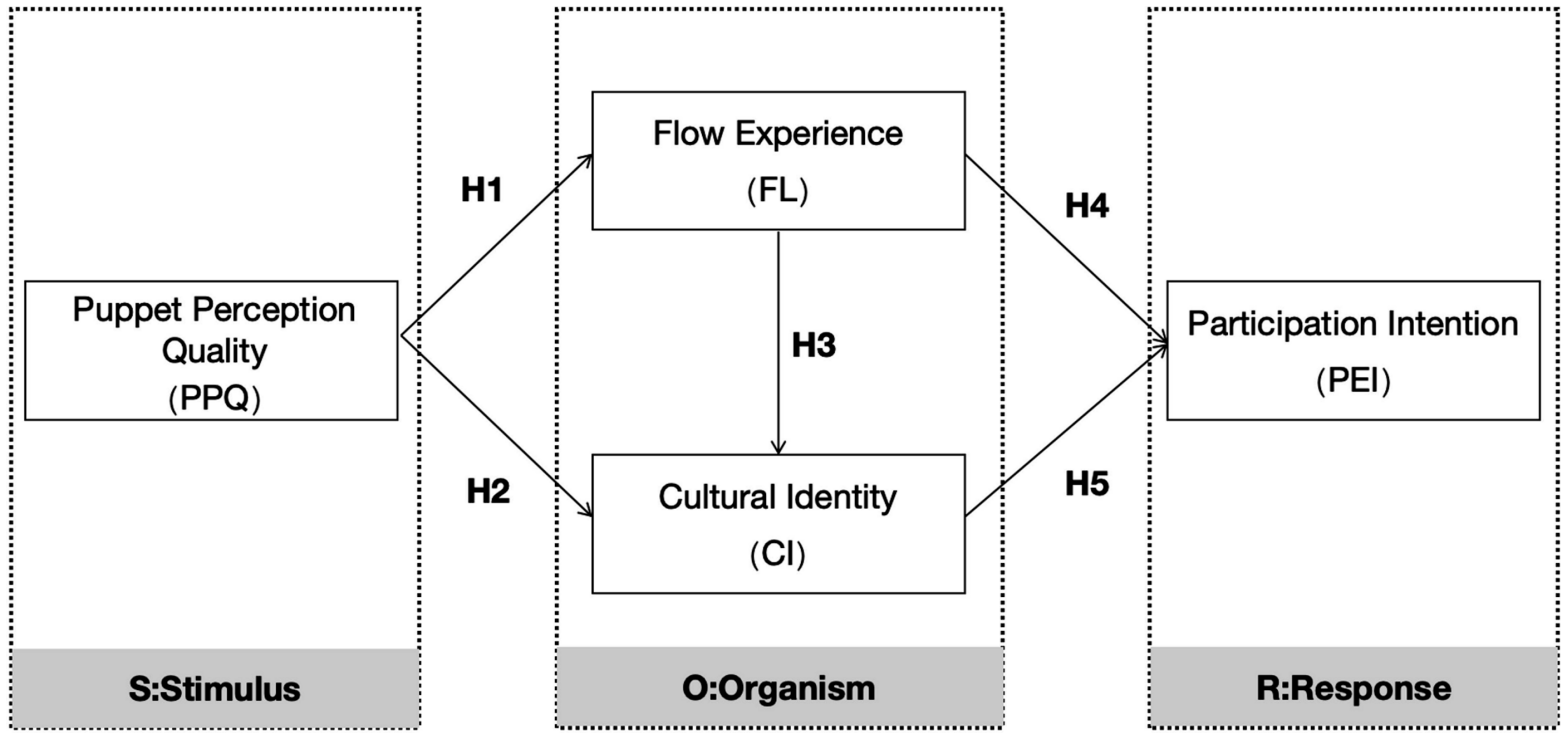
The proposed S-O-R theoretical framework for public participation intention in Liaoxi Puppet Opera.

By setting Flow Experience and Cultural Identity as dual-path variables at the organism level, this study reveals how the audience gradually sublimates into deep recognition of ethnic values in immersive aesthetic pleasure and ultimately leads to actual protection and participation behavior (R) (Figure 3). This logical closed loop not only improves the empirical rigor of the research but also provides a scientific path guidance for the transformation of endangered ICH from landscape display to endogenetic transmission.

### Public participation intention in ICH

The essential characteristics of ICH are its active and rheological (Hafstein, 2008). Since the adoption of the Convention for the Protection of ICH by UNESCO in 2003, the global paradigm of ICH protection has experienced a profound revolution from static materialized protection to living transmission (Kirshenblatt-Gimblett, 2014). The early protection model mostly focused on rescue conservation, that is, trying to solidify endangered art in a specific time and space through digital archiving, establishing museums, physical collection and other means (Kurin, 2007). This model can effectively prevent the physical death of skills in the short term, but its drawback is that it strips off the organic connection between the ICH and the social soil on which they depend for survival, making it a display that is otherwise (Arizpe, 2004). With the deepening of the research on heritage, the academic community has gradually realized that the essence of ICH lies not in the artefacts themselves, but in the social practice and community identity associated with them (Smith, 2006). As a result, Living Transmission has become the core paradigm of contemporary ICH protection. The paradigm emphasizes that ICH should be constantly re-created in the modern life situation, so that it’s social function has shifted from a single aesthetic display to the reconstruction of ethnic memory and the production of Cultural Identity (Bedjaoui, 2004). For performing arts such as Liaoxi puppetry with deep folk soil and exquisite skills, living transmission means that its performance should not only exist on a closed stage or video but should re-enter the aesthetic consumption and emotional life of the public through interaction with the audience, facilitating a transition from passive exhibition to active integration into modern life. Under the paradigm of living transmission, ICH is no longer a lonely struggle of the government or inheritors, but a kind of cultural governance with multi-party participation (Xia et al., 2020). Among them, the audience’s Participation Intention is regarded as the terminal variable that determines the non-replicability. In the situation of ICH protection, the Participation Intention not only includes the consumption motivation of watching performances but also covers deep protection behaviors such as recommending to others, learning relevant skills, and spreading ICH knowledge in social networks (Xiao et al., 2025).

Why does the Participation Intention have a central position? First, it is the primary predictor of ICH return of social functions. Local plays such as Liaoxi puppetry used to be the emotional connection of local society. Only when the contemporary public has a strong Participation Intention can this art form, which is marginalized by modern entertainment, regain the market vitality and realize the dual internal cycle of economy and culture. More importantly, with the support of the National Arts Fund of China and the joint efforts of the research team of the School of Arts of Northeast University, Liaoxi puppetry has made certain breakthroughs in digital protection, and the digital collection system, digital museum and intelligent identification system of Liaoxi puppetry have been established (Xie X. et al., 2024). However, the most core dilemma in inheritance is still the lack of participation in all dimensions, which seriously shackles its survival and development. Therefore, it is very important and significant to explore the psychological mechanism of the participation of Puppet Operas in Liaoxi. Secondly, the Participation Intention is the premise of Value Co-creation. The audience is no longer a passive recipient of information, and its feedback, evaluation and communication behavior itself are reshaping the contemporary value of ICH (Zou et al., 2025). When the public is willing to be present and advocate for the ICH, the ICH has the resilience to counterbalance the erosion of globalization (Zhao et al., 2025). Therefore, exploring the psychological causes that affect the Participation Intention in Liaoxi Puppet Operas is essentially to find the source of motivation for ICH to move from endangered to recovery.

### Hypothesis development

In the S-O-R framework, the visual aesthetic characteristic as an environmental stimulus is the initial factor that elicits the organism’s psychological response (Lavie and Tractinsky, 2004). According to the theory of flow, high-quality sensory clues can quickly occupy the individual’s attention resources, allowing them to withdraw from the surrounding environment and enter a state of immersion that is distorted by a sense of time (Reber et al., 2004). Many empirical studies have confirmed the positive driving effect of visual perception on Flow Experiences (Fang and Chung, 2025). For example, in consumer experience research, visual atmosphere has a positive impact on the Flow Experience, which shows that favorable visual perception can promote the formation of an immersive psychological state (Ettis, 2017). In addition, the study on aesthetic experience points out that when viewing works of art, when the audience is deeply immersed in visual perception activities, their experience has the characteristics like the flow of mind (Csikszentmihalyi and Robinson, 1990). Similarly, in the context of physical museums, He et al. (2018) pointed out that the visual presentation quality of the exhibits (such as layout, lighting, modelling) directly associated with the concentration and pleasure of visitors (He et al., 2018). For Liaoxi puppetry, the visual spectacle shown by its unique Pistol-grip control mechanism manipulation skills constitute high-intensity external stimulation. Kirillova et al. (2014) further emphasized that a high level of Aesthetic Quality can effectively reduce the self-awareness of individuals and promote them to enter a selfless psychological state (Kirillova et al., 2014). Therefore, when the audience perceives that the puppet performance is more exquisite and the visual presentation is more aesthetic, the more likely they are to have a deep immersive experience. Based on the theoretical and empirical foundations, this study proposes the following hypothesis.

*H1*: Puppet Perception is positively associated with Flow Experience.

Cultural Identity refers to the sense of belonging and psychological connection established by individuals through the sharing of symbols, practices and heritage with specific cultural groups (Hall, 1997). In the context of ICH, visual symbols and cultural clues in the performance are the carriers of collective memory and group identity (Geertz, 2017). According to the view of Social Semiotics, the visual presentation of ICH is not only an aesthetic object, but also a cultural text that can be decoded by the audience (Yaco and Ramaprasad, 2018). Cultural symbols are not only cognitively meaningful but also can connect viewers to cultural roots and shared values through emotional resonance (Barthes, 1977). Empirical research further substantiates the causal link between perceived quality and identity formation. Research on cultural and consumer psychology shows that visual symbols and cultural icons have the effect of enhancing group identity. They can evoke shared values and strengthen individual cultural belonging (Belk, 2013). In the study of heritage tourism and art participation, there is an empirical relationship between exposure to culturally significant visual stimuli and Cultural Identity (Lewicka, 2011). Jorgensen found that the symbolic expression of cultural objects and symbols can enhance the sense of social identity of community members, thus strengthening their connection with cultural traditions (Jorgensen and Stedman, 2001). Palmer pointed out in the study of heritage tourism that the core visual symbols are the key medium to awaken the sense of national identity (Palmer, 1999). In the study of ICH products, Zhang found that when consumers perceive that products have a high degree of Cultural Authenticity, their inner sense of group belonging will be significantly activated (Zhang et al., 2023). Similarly, Bonn et al. (2007) confirmed that the unique environmental cues of the heritage site can effectively elicit the emotional attachment and identity identification of tourists (Bonn et al., 2007). For Liaoxi puppetry, its realistic shape and embedded folk elements constitute a powerful cultural cue. When the audience recognizes these familiar geographical symbols in the puppet image, there will be a mirror effect, that is, seeing their own life and history in the artistic image. This perception process based on Self-Environment Congruity can quickly activate the latent collective memory, thus strengthening the deep identification with the local culture. Therefore, Puppet Perception is likely to enhance its Cultural Identity when the audience perceives and resonates with these familiar cultural clues. Based on the above theory and empirical evidence, this study puts forward the hypothesis.

*H2*: Puppet Perception is positively associated with Cultural Identity.

Within the Organism, the hedonistic emotional experience is often the prelude to deep value recognition. Immersion theory and experiential consumption theory point out that highly immersive experience can significantly reduce the psychological defense level of individuals and enhance their acceptance of cultural symbols and value connotations (Skadberg and Kimmel, 2004). When an individual enters a state of Flow, his cognitive resources are mainly concentrated in current activities, and emotional reactions dominate the evaluation process, thus promoting emotional connection and identity construction (Holt, 1995). Empirical research in cultural tourism and heritage consumption further demonstrates that immersive experiences effectively enhance cultural attachment and identity formation. Kim found that unforgettable and highly immersive cultural experiences help to strengthen the Cultural Identity and emotional connection of tourists (Kim et al., 2012); Taheri pointed out that in-depth experience participation can significantly improve emotional attachment and cultural loyalty (Taheri et al., 2014). Schouten confirmed in a study on extraordinary customer experience that when consumers experience a state of selfless immersion in an activity, they are more likely to have a deep emotional connection with the community or culture to which the activity belongs (Schouten et al., 2007). In the context of performing arts, the Flow Experience can promote the emotional resonance between the audience and the cultural narrative, so that aesthetic pleasure can gradually be transformed into symbolic self-identity (Pine and Gilmore, 1998). Through continuous immersion, the audience constantly internalizes the cultural significance carried by the performance and integrates it into the self-concept. Therefore, the Flow Experience plays a key psychological bridge between pleasant participation and deep cultural belonging. When the audience gets the ultimate Flow Experience by watching the Liaoxi Puppet Opera, this positive emotion will break their original cognitive stereotypes and make them feel psychologically close and open to the art form. When the audience has a highly immersive and pleasant experience, they are more likely to regard the art form as part of their own Cultural Identity, rather than simply entertainment consumption. Based on the above theory and empirical basis, this study puts forward the hypothesis.

*H3*: Flow Experience is positively associated with Cultural Identity.

According to Ajzen (1991) Theory of Planned Behavior (TPB), behavioral willingness is the most direct factor in predicting individual actual behavior (Ajzen, 1991). In the situation of experiential economy and hedonistic consumption, positive emotional experience plays a central role in shaping individual future participation in decision-making. From the perspective of Intrinsic Motivation Theory, the Flow Experience itself is a kind of self-rewarding (Csikszentmihalyi and Robinson, 1990). When individuals gain a sense of self-forgetting pleasure in activities, this positive emotional experience will form a Positive Reinforcement Mechanism (Deci and Ryan, 2000). Pleasant experiences will form positive emotional memories, prompting individuals to repeat similar behaviors to get psychological rewards again (Bagozzi, 1992). Many empirical studies show that the Flow Experience can significantly enhance the willingness to continue to use, the willingness to visit and the behavior of word-of-mouth communication. For example, Koufaris confirmed in consumer behavior research that the stronger Flow Experience, the higher the user’s satisfaction with the experience process, which in turn shows a stronger willingness to revisit (Koufaris, 2002); Novak pointed out that the Flow Experience in the online environment can improve users’ return visits (Novak et al., 2000). Wu and Li further verified in tourism research that immersive experience can strengthen tourists’ willingness to revisit and recommend (Wu et al., 2018). In the context of digital communication, this logic is also applicable. Chen and Lin’s empirical research in the live broadcast context found that Flow Experience significantly has a significant positive impact on users’ willingness to continue to use and social interaction (Chen and Lin, 2018). For Liaoxi puppetry, when the audience is attracted by the visual spectacle of the Pistol-grip control mechanism and enters a state of immersion, they will have a strong motivation to repeat this experience or share it with others to obtain the same psychological satisfaction again. In other words, the Flow Experience transforms heritage protection from a boring responsibility into an engaging pursuit. Therefore, the Flow Experience constitutes an important psychological motive to promote the Participation Intention. Based on this, this study puts forward the hypothesis.

*H4*: Flow Experience is positively associated with Participation Intention.

Unlike a brief pleasure brought by Flow Experience, Cultural Identity provides a more lasting and deeper behavioral primary predictor. Social Identity Theory believes that individuals tend to define self-identity through group belonging and maintain group values through behaviors that are favorable to the group (Turner et al., 1979). When individuals form a high degree of identification with a cultural group, group goals will be internalized into personal responsibilities and further transformed into continuous behavioral commitment. In the field of ICH protection, Cultural Identity is the key variable to transform an audience member into a guardian. A high degree of Cultural Identity can enhance an individual’s sense of responsibility, emotional attachment and moral obligation, thus promoting their participation in cultural protection and dissemination (Ashworth et al., 2007). From the perspective of self-consistency theory, when cultural symbols are highly compatible with individual self-concepts, their participatory behavior will be further strengthened (Sirgy et al., 1997).

Many empirical studies support a positive relationship between Cultural Identity and Participation Intention. For example, Chhabra found that cultural heritage identity can significantly predict the willingness of tourists to revisit and support (Chhabra et al., 2003); Su confirmed that Cultural Identity can enhance the audience’s Participation Intention in ICH protection and promotion activities (Su et al., 2018); Zhang also pointed out that Identification is an important factor in predicting the behavioral intention of ICH products (Zhang et al., 2023). In the context of endangered performing arts such as Liaoxi puppetry, when the audience forms a strong sense of identity that this is our cultural heritage, this emotional bond will be further transformed into the intrinsic motivation of continuous participation, active dissemination and voluntary protection. Therefore, Cultural Identity constitutes an important emotional motivator that promotes the Participation Intention. Based on the above theory and empirical basis, this study puts forward the hypothesis.

*H5*: Cultural Identity is positively associated with Participation Intention.

## Methods and methodology

### Stimulating material design and ecological validity control

To ensure the authority and high ecological validity of experimental stimulation materials, this study adopts a questionnaire survey based on real situations. Before filling out the questionnaire, all respondents were asked to watch a 2-min high-definition video clip of Long Silk Dance (Chang Chou Wu), a classic play of Liaoxi puppetry. To strictly control the exposure time and ensure full engagement, the online survey platform was programmed with a mandatory 120-s time lock, preventing respondents from skipping the video or proceeding to the questionnaire before the video had finished. It is especially worth noting that the video material was not sourced from the Internet; rather, it was an exclusive recording commissioned by the research team and performed live by Master Wang Na, the national ICH inheritor of Liaoxi Puppet Opera. To supplement the visual details, respondents were also asked to view 8 high-definition images depicting puppet characters and performance scenes. These 8 images were personally selected by the inheritors of this ICH and were further cross validated by an expert panel consisting of three scholars in traditional theatrical arts and ICH preservation to ensure their representativeness and ecological validity. They capture the most representative movements, costume motifs and facial features, and best encapsulate the visual essence of Liaoxi Puppet Opera. Additionally, a manipulation check was conducted immediately after the stimuli presentation. Respondents were required to answer a simple screening question regarding a core visual element presented in the materials to verify their attentive observation. Only those who passed this manipulation check were retained in the final dataset. These constitute valuable primary source materials provided exclusively by the inheritor. These high-standard stimulating materials aim to restore the visual aesthetics and performance skills of Liaoxi puppetry in an all-round and high-fidelity way, minimize the deviation between the experimental environment and the real viewing experience, and ensure that respondents can answer based on the most realistic audio-visual experience.

### Measurement scale development

A measurement scale is developed based on the four core concepts of the S-O-R theoretical framework, and a structured questionnaire presented in a statement way is used for measurement. In the research model, the independent variable (S) is Puppet Perception (PPQ). The variable (O) includes Flow Experience (Fl) and Cultural Identity (CI), in which Cultural Identity is conceptualized as a comprehensive variable containing cognition and emotion. The dependent variable (R) is Participation Intention (PEI). Given the high alignment between the core dimensions of the established scales and the objectives of this study, these instruments were adapted to fit the specific context of Liaoxi Puppet Opera. Specifically, the items from the original scales were employed to accurately assess the audience’s psychological states and behavioral intentions induced by visual aesthetic stimuli. When adjusting the questionnaire questions, this study has borrowed the existing scale and optimized language readability to adapt to the specific characteristics of ICH protection. To ensure the cross-cultural validity of the survey tools, a rigorous translation and back-translation procedure (Brislin, 1970) was employed. Two bilingual researchers independently translated the original English items into Chinese, and a third bilingual researcher back-translated them into English to ensure semantic equivalence. The adapted scale was then subjected to expert validation by a panel of five experts who evaluated the content validity and clarity of the items. Subsequently, a pilot test was conducted with a sample of 100 respondents (*N* = 100) to verify the reliability and readability of the questionnaire before the formal survey. The full list of measurement items and their sources is provided in the Supplementary Material.

All latent variable items utilized a 5-point Likert scale (1 = strongly disagree, 5 = strongly agree). Respondents were asked to check the corresponding options according to their true feelings after watching the video. The specific scale sources are as follows: Puppet Perception (PPQ) was measured using five items adapted from (Kirillova et al., 2014), focusing on visual presentation and performance skills. Flow Experience (Flow) was measured using five items adjusted based on the flow state scales of (Koufaris, 2002) and (Novak et al., 2000). Regarding the operationalization of Cultural Identity (CI), it was measured using six items. In the context of endangered ICH, Cultural Identity is conceptualized not merely as a demographic label, but as a dynamic, multidimensional socio-psychological construct encompassing both cognitive group belongingness and affective evaluation. To capture this accurately, four items regarding belongingness were adapted from organizational identification literature (Mael and Ashforth, 1992) and heritage studies (Zhang et al., 2023) to reflect the audience’s cognitive overlap with the regional culture represented by Liaoxi puppetry. The remaining two items regarding affective attitude were adapted from established attitudinal scales (Ajzen, 1991) to capture the deep psychological attachment and positive evaluation toward safeguarding this cultural root. Participation Intention (PEI) was measured using four items developed based on Zeithaml et al. (1996) and Su and Swanson (2020).

Additionally, reverse-coded items and attention checks were embedded in the questionnaire to control for common method bias and careless responses.

### Data collection

The data collection process of this study is divided into two stages: Pilot Test and Formal Survey, both of which were carried out through the online survey platform Wenjuanxing. In strict accordance with the ethical guidelines for human subjects research and under the formal approval of the Ethics Committee of Northeastern University, the first page of the questionnaire was set up with an informed consent form to clearly inform the respondents of the academic purpose of the research, the principle of anonymity and the right to withdraw at any time. Only the respondents who choose “agree” can enter the follow-up answer session. The questionnaire mainly collects the demographic information of the respondents (such as gender, age, education) and ICH contact characteristics (such as viewing frequency).

To test the credibility of the measuring tool, the research team first conducted a small-scale pilot test in March 2026. Based on the feedback of pre-research, the wording of some questions was semantically optimized. The official survey was conducted from 18 March to 10 April 2026, and questionnaires were distributed through social media platforms. Given the specific characteristics of ICH engagement and the exploratory nature of the theoretical model, a non-probability sampling strategy—specifically convenience sampling combined with the snowball method—was employed. This approach is widely accepted in heritage studies and behavioral research when a complete sampling frame is unavailable (Calder et al., 1981). Although adults aged 18 years and above were eligible to participate, the present study primarily focused on younger and digitally engaged audiences. Younger generations are increasingly exposed to cultural content through online platforms and represent an important target group for contemporary ICH communication and revitalization. Therefore, the online distribution strategy intentionally emphasized individuals who actively use digital media, which resulted in a sample largely composed of younger respondents. Consequently, the findings should be interpreted primarily in relation to younger audiences rather than the general population. To strictly control the data quality, two reverse scoring questions and attention test questions were randomly inserted into the questionnaire to identify and eliminate invalid answers. A total of 518 questionnaires were collected in this survey. After strict screening, after eliminating invalid samples with insufficient time to answer, showing regular answers (Straight-lining) or failure to pass the attention test, 440 valid questionnaires were finally obtained, with an effective recovery rate of 85%.

### Data analysis

Data analysis was carried out using IBM SPSS 27.0 and AMOS 26.0 in two main stages. In the first stage, SPSS 27.0 was used to generate descriptive statistics to summarize the demographic characteristics of the respondents. At the same time, Cronbach’s *α* coefficients were calculated to assess the internal consistency and reliability of each latent construct. In the second stage, structural equation modeling (SEM) was performed following the two-step procedure proposed by Anderson and Gerbing (Anderson and Gerbing, 1988). First, confirmatory factor analysis (CFA) was conducted in AMOS 26.0 to evaluate the measurement model, with particular attention paid to convergent and discriminant validity. After an acceptable measurement model was established, the structural model was estimated to test the proposed hypotheses and path relationships. It is important to note that while the S-O-R framework provides a theoretical ordering for the variables, the cross-sectional nature of the survey data means that true causality cannot be inferred. Therefore, the structural paths tested in this study represent theoretically informed sequential associations.

## Results

### Common method bias test

As the data in this study were collected through self-reported questionnaires at a single time point, the possibility of common method bias (CMB) cannot be completely ruled out. Therefore, Harman’s single-factor test was applied as a preliminary diagnostic procedure. Specifically, all measurement items, including those for Puppet Perception, Flow Experience, Cultural Identity, and Participation Intention, were entered into an unrotated exploratory factor analysis (EFA). The results showed that the first principal component accounted for 17.77% of the total variance (Table 1), which is substantially lower than the commonly suggested threshold of 50% (Podsakoff et al., 2003). To address the limitations of Harman’s test and provide stronger robustness checks, we further assessed CMB through the full collinearity Variance Inflation Factor (VIF) approach, which is recognized as a highly rigorous and conservative test (Kock, 2015), Following this procedure, all latent constructs were regressed against a randomly generated dummy variable to compute the full collinearity VIFs. The resulting VIF values for Puppet Perception, Flow Experience, Cultural Identity, and Participation Intention were 1.686, 1.583, 2.127, and 1.569, respectively. All values are well below the stringent threshold of 3.3, indicating that the model is free from severe common method bias.

**Table 1:** Exploratory factor analysis results (*N* = 440).

Items	Factor 1	Factor 2	Factor 3	Factor 4
Cultural identity				
CI2	0.803			
CI4	0.735			
CI3	0.72			
CI1	0.689			
CI5	0.657			
CI6	0.554			
Puppet perception				
PPQ5		0.763		
PPQ3		0.75		
PPQ2		0.739		
PPQ4		0.73		
PPQ1		0.722		
Flow experience				
FL1			0.808	
FL4			0.782	
FL5			0.75	
FL2			0.692	
FL3			0.682	
Participation intention				
PEI1				0.801
PEI2				0.799
PEI3				0.783
PEI4				0.658
Eigenvalues	8.47	1.87	1.59	1.26
Variance explained %	17.77%	16.91%	16.28%	15.02%
Cumulative %	65.99%			
KMO measure	0.947			
Bartlett’s test	*p* < 0.001 (*χ*^2^ = 5456.16, *df* = 190)			

Furthermore, an examination of the correlation matrix indicated that all inter-construct correlation coefficients remained below 0.90 (Bagozzi et al., 1991). The highest observed correlation was 0.618 (Table 2). The excellent fit of our multi-factor confirmatory factor analysis (CFA) measurement model demonstrates strong discriminant validity among the constructs, reconfirming that a single method factor does not dominate the data structure. Taken together, these results suggest that the constructs in this study are sufficiently distinct, and that common method bias is unlikely to pose a serious threat to the validity of the findings.

**Table 2:** Descriptive statistics and correlation matrix.

Constructs	Mean	SD	Skewness	Kurtosis	1	2	3	4
Puppet perception (S)	3.99	0.83	−1.2	1.28	**0.740**			
Flow experience (O)	3.67	0.86	−0.63	−0.04	0.460**	**0.748**		
Cultural identity (O)	3.94	0.84	−0.96	0.42	0.618**	0.557**	**0.753**	
Participation intention (R)	3.74	0.89	−0.69	−0.03	0.433**	0.485**	0.560**	**0.762**

*N* = 440. **p < 0.01. Diagonal elements (in bold) represent the square root of the Average Variance Extracted (AVE). Off-diagonal elements represent the correlations between constructs.

### Descriptive analysis

A total of 440 valid questionnaires were obtained in this study. The demographic characteristics of the respondents are summarized in Table 3. In terms of gender, female participants slightly outnumbered male participants, accounting for 57% (*N* = 251) of the sample, while males represented 43% (*N* = 189). In terms of age, the sample was mainly composed of younger respondents. Participants aged between 18 and 35 formed the largest group, comprising 71.1% (*N* = 313), followed by those aged 35 to 60 (22.3%). We acknowledge that the 35–60 age bracket is relatively broad, which represents a limitation in the initial questionnaire design. However, this skewed distribution primarily reflects the reality of the online survey distribution strategy (via social media platforms) and the greater participation of digitally connected populations in web-based surveys. Therefore, the present sample should not be interpreted as empirical evidence that younger generations inherently constitute the primary audience of digitalized ICH. Nevertheless, younger audiences remain a crucial demographic for contemporary ICH revitalization, as they represent the future consumers, active participants, and potential inheritors of cultural heritage. Concerning educational background, more than half of the respondents (56%) reported holding a bachelor’s degree, and approximately 22% had completed postgraduate studies (Master’s or PhD). This suggests that the sample generally had a relatively high level of educational attainment and possessed adequate comprehension to accurately respond to the questionnaire. In terms of occupation, full-time employees (47.3%) and students (41.6%) constituted the two largest groups in the sample. Overall, while the sample is inherently skewed toward a younger, digitally engaged demographic due to the online data collection method, the respondents’ diverse occupational and educational backgrounds provided a sufficient basis for exploring the theoretical model within this specific context.

**Table 3:** Demographic characteristics of participants (*N* = 440).

Characteristic	Category	Frequency (*N*)	Percentage (%)
Gender	Male	189	43%
	Female	251	57%
Age	18–35	313	71.1%
	35–60	98	22.3%
	60+	29	6.6%
Education	Secondary education	97	22%
	Undergraduate	246	56%
	Postgraduate (Master/PhD)	97	22%
Occupation	Student	183	41.6%
	Full-time job	208	47.3%
	Part-time job	32	7.3%
	Unemployed	17	3.8%

### Measurement model assessment

To examine the structural validity of the latent variables, exploratory factor analysis (EFA) was first carried out using IBM SPSS 27.0. As shown in Table 1, the KMO value reached 0.947, which is far above the excellent level of 0.80 (Kaiser, 1974). Meanwhile, Bartlett’s Test of Sphericity was statistically significant (*χ*^2^ = 5456.16, *df* = 190, *p* < 0.001), indicating that the dataset was appropriate for further factor analysis. Principal component analysis (PCA) with Varimax rotation was then applied. Four common factors with eigenvalues greater than 1.0 were extracted, which were generally consistent with the proposed dimensions, namely Cultural Identity, Puppet Perception, Flow Experience, and Participation Intention. These four factors together explained 65.99% of the total variance, exceeding the 60% threshold suggested for social science research. In addition, the factor loadings of all measurement items ranged from 0.554 to 0.808, which were higher than the acceptable minimum of 0.50 (Hair et al., 2009). No serious cross-loadings were observed among the items. Overall, the results provide preliminary support for the structural validity of the measurement scale.

Subsequently, Confirmatory Factor Analysis (CFA) was conducted using IBM SPSS AMOS 26.0 to further examine the reliability and validity of the measurement model (Table 4). In terms of reliability, the Composite Reliability (CR) values of all constructs ranged from 0.846 to 0.887, which were clearly above the recommended threshold of 0.70 (Fornell and Larcker, 1981), indicating satisfactory internal consistency of the scale.

**Table 4:** Results of reliability and validity analysis.

Construct/item	Standardized loading (SFL)	Composite reliability (CR)	Average variance extracted (AVE)
Puppet perception (PPQ)		0.858	0.548
PPQ1	0.704		
PPQ2	0.74		
PPQ3	0.777		
PPQ4	0.742		
PPQ5	0.737		
Flow experience (Flow)		0.864	0.56
FL1	0.748		
FL2	0.833		
FL3	0.703		
FL4	0.738		
FL5	0.713		
Participation intention (PEI)		0.846	0.58
PEI1	0.776		
PEI2	0.808		
PEI3	0.765		
PEI4	0.692		
Cultural identity (CI)		0.887	0.567
CI1	0.737		
CI2	0.722		
CI3	0.812		
CI4	0.795		
CI5	0.755		
CI6	0.691		

SFL, standardized factor loading; CR, composite reliability; AVE, average variance extracted. All loadings are significant at *p* < 0.001. Acceptable thresholds: SFL > 0.50, CR > 0.70, AVE > 0.50.

About convergent validity, two main criteria were considered. First, all standardized factor loadings ranged between 0.691 and 0.833, exceeding the minimum acceptable level of 0.50, and all loadings were significant at the *p* < 0.001 level. Second, the Average Variance Extracted (AVE) values varied from 0.548 to 0.580, which were higher than the suggested cutoff value of 0.50. Taken together, these results indicate that the latent constructs can explain a sufficient proportion of variance in their observed indicators, providing evidence of adequate convergent validity.

Finally, discriminant validity was evaluated using the Fornell–Larcker criterion (Fornell and Larcker, 1981). As shown in Table 2, the square roots of the AVE values for each construct (reported on the diagonal in bold) ranged from 0.740 to 0.762. In all cases, these values were higher than the corresponding inter-construct correlations in the same rows and columns, with the maximum correlation coefficient being 0.618. This suggests that, although the constructs are theoretically related, they remain sufficiently distinct at the statistical level.

In addition, descriptive statistics were examined to assess the normality of the data. The absolute values of skewness ranged from 0.63 to 1.20, while kurtosis values varied between 0.03 and 1.28. All these values fell well within the acceptable range of ±2 (Gravetter et al., 2021), indicating that the data did not deviate substantially from normality and were appropriate for Maximum Likelihood estimation.

### Structural model assessment

After establishing the reliability and validity of the measurement model, Structural Equation Modeling (SEM) was applied to examine the proposed research hypotheses. The model fit indices are reported in Table 5. The ratio of chi-square to degrees of freedom (*χ*^2^/*df*) was 2.293, which falls below the commonly accepted threshold of 3.0. The RMSEA value was 0.054, indicating an acceptable level of approximation.

**Table 5:** Fit Indices for the structural model.

Model	*χ*^2^	*df*	*χ*^2^/*df*	GFI	AGFI	TLI	CFI	RMSEA
Measurement model	377.783	164	2.304	0.917	0.894	0.946	0.953	0.054
Structural model	378.376	165	2.293	0.917	0.895	0.946	0.953	0.054
Recommended threshold	—	—	<3.0	> 0.90	>0.80	>0.90	>0.90	<0.08

*N* = 440. *χ*^2^ = Chi-square; *df* = Degrees of freedom; GFI, goodness of fit index; AGFI, adjusted goodness of fit index; TLI, Tucker–Lewis index; CFI, comparative fit index; RMSEA, root mean square error of approximation.

About incremental fit measures, the CFI (0.953), TLI (0.946), and GFI (0.917) all exceed the recommended cutoff value of 0.90 (Hu and Bentler, 1999). Taken together, these results suggest that the proposed model fits the observed data well and provides adequate support for subsequent hypothesis testing.

**Figure 4:**
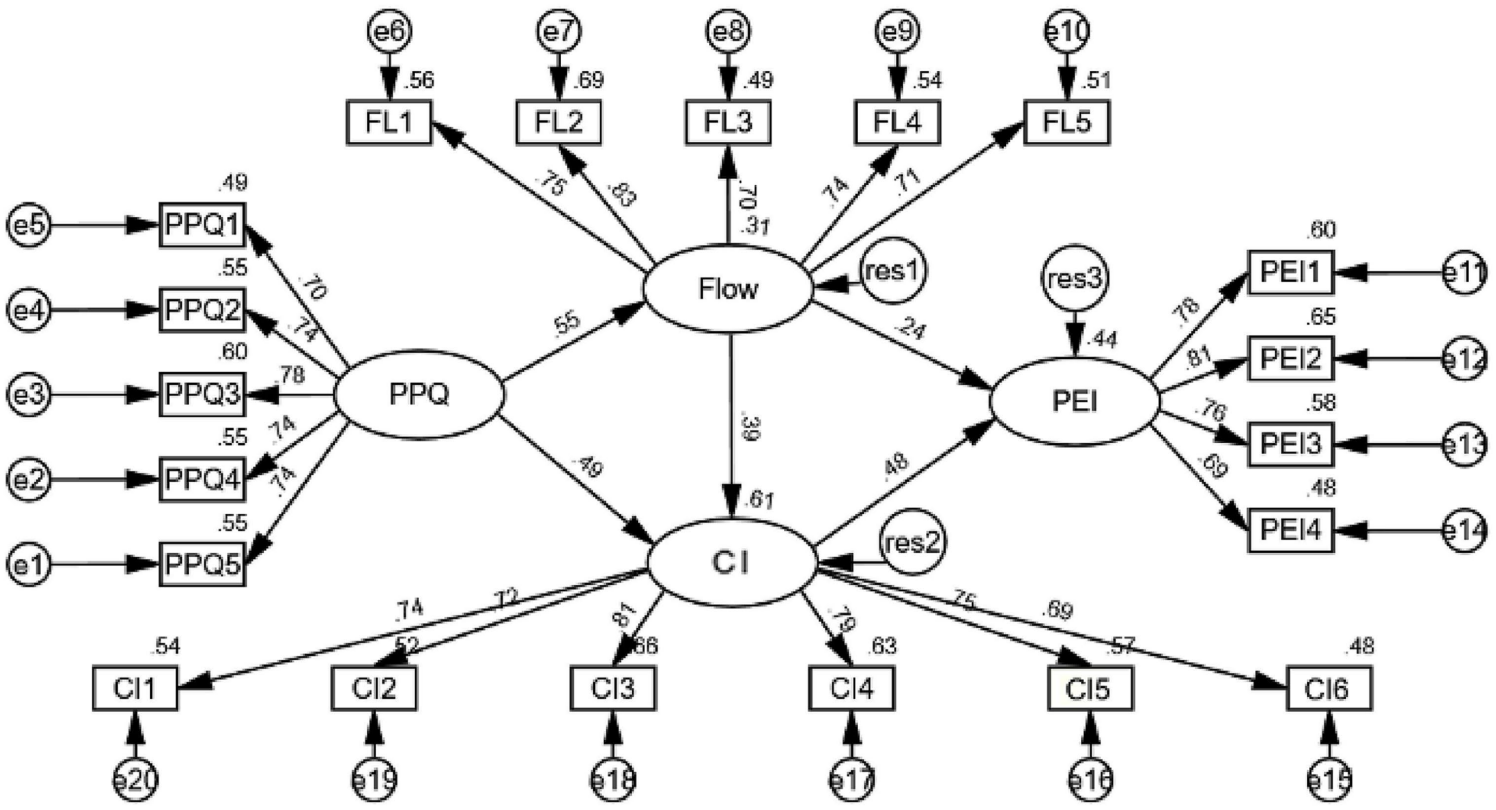
The final structural model (standardized estimates).

The results of the path analysis and hypothesis testing are shown in Table 6 and Figure 4. First, Puppet perception, as an environmental stimulus, has a significant positive association with the organismic response: it significantly predicts the Flow Experience (*β* = 0.55, *p* < 0.001) and Cultural Identity (*β* = 0.49, *p* < 0.001), thus supporting H1 and H2. This shows that the unique visual aesthetics of Liaoxi puppetry is a key antecedent for the psychological immersion and cultural resonance of the audience.

**Table 6:** Results of structural model and hypothesis testing.

Hypothesis	Path relationship	B	*β*	S.E.	C.R. (*t*)	*p*	Result
H1	Puppet Perception→Flow Experience	0.548	0.55	0.058	9.362	***	Supported
H2	Puppet Perception→Cultural Identity	0.478	0.49	0.058	8.288	***	Supported
H3	Flow Experience→Cultural Identity	0.381	0.39	0.054	7.01	***	Supported
H4	Flow Experience→Participation Intention	0.273	0.24	0.076	3.606	***	Supported
H5	Cultural Identity→Participation Intention	0.559	0.48	0.083	6.77	***	Supported

***p < 0.001. Unstd. Estimate, unstandardized regression weights; S.E., standard error; C.R., critical ratio.

Secondly, within the organism, the Flow Experience is significantly positively associated with Cultural Identity (*β* = 0.39, *p* < 0.001) and supports H3. This shows that a pleasant aesthetic experience can be transformed into deep emotional identity. Finally, in terms of the relationship between of the organism on the response, the Flow Experience (*β* = 0.24, *p* < 0.001) and Cultural Identity (*β* = 0.48, *p* < 0.001) both are significantly positively associated with the Participation Intention, supporting H4 and H5.

To explicitly test whether the predictive strength of Cultural Identity on Participation Intention was significantly stronger than that of Flow Experience, a constrained model comparison was conducted using AMOS. Specifically, an unconstrained baseline model was compared with a constrained model in which the path coefficients for Flow→PEI and CI→PEI were forced to be equal. The results revealed a significant deterioration in model fit for the constrained model (Δ*χ*^2^(1) = 8.635, *p* < 0.01). This formal statistical test confirms that the standardized path coefficient of Cultural Identity (0.48) is indeed significantly higher than that of Flow Experience (0.24). This reveals a core discovery: although the pleasurable Flow Experience can influence behavioral intention, awakening the audience’s deep Cultural Identity is the strongest primary predictor to promote their participation in behavior.

To further evaluate how well the proposed model explains the key variables, this study examined the squared multiple correlations (*R*^2^) of the endogenous constructs (Table 7). The results indicate that the model explains 60.8% of the variance in Cultural Identity (*R*^2^ = 0.608), 44.3% in Participation Intention (*R*^2^ = 0.443), and 30.7% in Flow Experience (*R*^2^ = 0.307). In other words, a relatively large proportion of the variance in these core variables can be accounted for by the current model. According to Chin, *R*^2^ values around 0.67, 0.33, and 0.19 are generally regarded as substantial, moderate, and weak, respectively (Chin, 1998). From this perspective, the explanatory power for Cultural Identity in this study is close to the substantial level, while Participation Intention and Flow Experience reach a moderate level. Although the predictive strength differs across constructs, the overall results suggest that the S-O-R framework adopted in this study is effective in capturing the main psychological mechanisms underlying audience participation in ICH activities.

**Table 7:** Variance Explained (R^2^) of Endogenous Constructs.

Endogenous construct	*R*^2^ (squared multiple correlation)	Predictive power
Flow experience	0.307	Moderate
Cultural identity	0.608	Substantial
Participation intention	0.443	Moderate to substantial

*R*^2^ values of 0.19, 0.33, and 0.67 are described as weak, moderate, and substantial, respectively (Chin, 1998).

### Mediation effect analysis

To further clarify the intrinsic conduction mechanism of the S-O-R framework, this study adopts the Bootstrap method (setting 5,000 samples, 95% deviation correction confidence interval) to test the mediating roles of organismic variables. The analysis results are shown in Table 8.

**Table 8:** Mediation effects testing results.

Mediation path description	Point estimate (*β*)	95% confidence interval (BC) lower - upper	Result
Via flow experience (PPQ → Flow → PEI)	0.15	[0.098, 0.213]	Significant
Via cultural identity (PPQ → CI → PEI)	0.267	[0.195, 0.348]	Significant
Serial mediation (PPQ → Flow →CI → PEI)	0.117	[0.075, 0.169]	Significant
Total indirect effect	0.534	[0.442, 0.635]	Significant

The results show that the Total Indirect Effect of Puppet perception on the Participation Intention is significant (*β* = 0.534, 95% CI = [0.442, 0.635]). Specifically, the analysis revealed three unique conduction paths: the intermediary role through the Flow Experience: the indirect path through the Flow Experience is significant (*β* = 0.150, 95% CI = [0.098, 0.213]). This confirms that the aesthetic quality of puppetry partly transmits its influence on behavioral will by experiencing a state of psychological immersion. The intermediary role through Cultural Identity: the indirect path through Cultural Identity is also significant (*β* = 0.267, 95% CI = [0.195, 0.348]). The key is that the effect of this path is significantly greater than that of the Flow path, which shows that awakening cultural resonance is the primary psychological bridge between environmental stimulation and participation in behavior. Chain intermediary role: The chain intermediary path of Puppet Perception→Flow Experience→Cultural Identity→Participation Intention has also proved to be remarkable (*β* = 0.117, 95% CI = [0.075, 0.169]). This discovery highlights a sequence mechanism, that is, the hedonic Flow Experience not only directly promotes the will but also acts as a facilitator to further stimulate deep Cultural Identity. Given that the 95% confidence interval of all three paths does not contain 0, the intermediary role of Flow Experience and Cultural Identity is empirically supported. These findings verify that the Liaoxi puppetry promotes participation through the dual path mechanism of enjoyment experience and cultural awakening, of which the latter plays a more dominant role.

## Discussion

Based on the S-O-R framework, this study empirically tests the psychological mechanism behind the public’s participation in the protection of puppetry in Liaoxi. The research results support all hypothetical relationships and reveal the dual-path mechanism of the synergy of aesthetic experience and cultural cognition. The specific path coefficient and intermediary associations provide profound theoretical insights into the consumption behavior of endangered ICH.

### Material anchor effect: visual aesthetics as a dual antecedent

The study found that Puppet Perception significantly predicted the Flow Experience (H1) and Cultural Identity (H2). This discovery confirms Kirillova point of view (Kirillova et al., 2014), that is, visual aesthetics is the primary sensory interface of consumer judgement. However, this study expands their conclusions and suggests that for endangered ICH, the function of aesthetics goes beyond simple sensory stimulation. The unique Pistol-grip control mechanism manipulation and Human-puppet co-performance modelling of Liaoxi puppetry serve as the Material Anchors described by Hutchins (2005)—that is, the physical object of the integration of stable concepts (Hutchins, 2005). In addition, it is crucial to realise that the visual forms of Liaoxi Puppet Opera, such as specific clothing patterns, symbolic performance elements and regional visual clues - are far more than just aesthetic stimulation. As evidenced by Li S. et al. (2024) and Li W. et al. (2024) in his research on artistic heritage and perception evaluation, such visual images are usually used as deep cultural coding, reflecting ritual memory and collective belonging (Li W. et al., 2024). Against the background of the Liaoxi Corridor, these visual elements exist as carriers of the meaning of social inheritance and the meaning and kinship-ritual significance. By transcending pure perceptual stimulation, our research results show that Puppet Perception acts as a bridge connecting modern audiences with their ancestral roots and shared regional identity. This kinship-ritual perspective helps explain why cultural identity exerts such a dominant influence: the puppet is not just an artistic object, but a visual manifestation of communal memory and collective soul.

Specifically, the Pistol-grip control mechanism technique breaks through the physical limitations of traditional stick-headed puppets, creating a high-intensity visual novelty, and quickly capturing the audience’s immediate attention. This kind of visual impact creates an aesthetic distance, allowing the audience to move away from reality and enter a state of Flow Experience. At the same time, the visual symbols embedded in the clothing act as symbolic clues, which activates the audience’s collective memory and directly linked to Cultural Identity. This discovery theoretically enriches the embodied cognition perspective in heritage research (Guo and Dechsubha, 2025), showing that ICH aesthetics has the essence of double coding: it is both encoded into the sensory pleasure of the individual and the cultural totem of the group. Unlike the aesthetics in general tourism landscapes that mainly serve hedonism (Breiby and Slåtten, 2018), the aesthetics of Liaoxi puppetry is the carrier of deep cultural significance.

### From immersion to identity: flow as a psychological facilitator

A novel contribution of this study is to provide support for a sequential association pattern consistent with the proposed framework (Puppet Perception**→**Flow Experience**→**Cultural Identity**→**Participation Intention) and support for H3. This shows that the Flow is not only an isolated state of pleasure, but also a psychological facilitator of Cultural Identity. This discovery challenges the traditional view that entertainment experience and serious cultural cognition are two separate areas (Bartsch and Hartmann, 2017). In the context of traditional ICH, modern audiences (especially young people) often face cognitive barriers due to the ancient sense of art forms. The Flow Experience characterized by high concentration and loss of self-consciousness (Csikszentmihalyi, 1988) effectively reduces this psychological Defense (Hamari et al., 2016). When the audience is immersed in the exquisite skills of puppetry, the stereotype of boring is deconstructed. According to the theory of cognitive evaluation, this positive emotional state will cultivate the inner motivation to understand the object, thus creating an open psychological state for the implantation of cultural values (Deci and Ryan, 2000). This indicates that edutainment can be effective cognitive path: people must first enter the experience (Flow) before they can embrace the spirit (Identification). This result is also consistent with the study of Skadberg and Kimmel (2004), who believe that Flow can promote learning, but this study goes further and specifically links flow to the formation of Cultural Identity, not just general knowledge acquisition.

### Eudaimonic over hedonic: the logic of cultural stewardship

The most critical theoretical contribution of this study is to reveal that the influence of Cultural Identity on Participation Intention is twice that of the Flow Experience. This sharp contrast highlights the unique behavioral logic of endangered heritage. In general consumer behavior, Hedonic Motivation (pleasure-seeking) tends to dominate (Hirschman and Holbrook, 1982). However, this study indicates that in the endangered field of ICH, Eudaimonic Motivation (meaning-seeking) prevails. The audience’s intention to participate comes not more from the short-lived fun brought by the performance, but from a deep sense of Cultural Stewardship a moral obligation to preserve one’s own roots. As Bhattacharya and Sen pointed out in their identity theory (Bhattacharya and Sen, 2003), when consumers’ self-concepts overlap with the organization or culture, they become their defenders. This study provides empirical support for this theory in the context of ICH, showing that although the Flow Experience is the bait to attract attention (Harris et al., 2019), Cultural Identity is the anchor to ensure long-term investment (Zhang et al., 2020). This means that the revival of Liaoxi puppetry fundamentally depends on awakening the cultural genes in the audience, not just providing sensory stimulation. This pulls the paradigm of ICH protection back from pan-entertainment to value awakening (Yang et al., 2021).

## Implications

### Theoretical implications

This study has made three substantial theoretical contributions to the protection of ICH and consumer behavior literature. First, this study goes beyond simply applying the established S-O-R framework to a new context by theorizing and verifying a sequential gateway mechanism. While previous environmental psychology studies often treat multiple organismic states (O) as parallel or competing, this study reveals that in the context of ancient and potentially distant endangered art, hedonic immersion (Flow) serves as a prerequisite for eudaimonic resonance (Cultural Identity). In the past, environmental psychology studies usually regarded Organism (O) as a single or general emotional state (such as pleasure or awakening), but this study deconstructs it into two unique but coordinated dimensions: emotional dimension (Flow Experience) and cognitive dimension (Cultural Identity). By verifying the chain intermediary path (Stimulus→Flow→Identity→Response), this study builds a theoretical bridge between environmental psychology and heritage research. It empirically proves that for endangered art, external artistic stimulation will not be directly transformed into behavioral commitment but requires a complex psychological transformation process - from sensory immersion to cognitive resonance. This provides a more refined theoretical perspective for understanding how external artistic stimuli are internalized into behavioral commitment. Secondly, this study reconstructs the motivation hierarchy of ICH consumption and provides a critical boundary condition for the entertainment-first paradigm frequently emphasized in general tourism literature. Existing tourism and leisure literature usually emphasizes that the pleasure experience is the ultimate primary predictor of tourist behavior. However, the empirical evidence of this study shows that in the specific situation of endangered heritage, the influence of realism motivation (Cultural Identity) on participation intention is significantly stronger than the motivation for pleasure (Flow Experience). This discovery theoretically distinguishes heritage protection from general tourism consumption. It redefined the core primary predictor of ICH protection as self-realization and cultural belonging, rather than mere sensory satisfaction, indicating that the endangered state of heritage elicits a unique moral obligation beyond simple happiness. Third, this study enriches the academic discussion on edutainment through empirical verification. By identifying that the Flow Experience is a key explanatory pathway associated with Cultural Identity, this study solves the long-term theoretical tension between popularization and desacralization. The results show that high-quality immersive experience is a psychological pathway to reduce cognitive barriers, allowing deep cultural values to penetrate. This means that entertainment and seriousness are not binary oppositions, but a continuous stage in the process of psychological acceptance. The Flow Experience acts as a facilitator for the modern audience’s indifferent attitude towards ancient art, making it possible to implant subsequent Cultural Identity.

### Practical implications

The results of the management revelation study provide actionable management insights for museum curators, ICH inheritors and cultural policymakers who aim to revitalize Liaoxi puppetry. First, given that Puppet Perception functions as the initial elicitor of subsequent psychological and behavioral responses, practitioners should place greater emphasis on the visual presentation of core artistic features. Traditional display methods based mainly on static exhibitions may limit audience engagement. Therefore, museums and cultural institutions are encouraged to incorporate digital tools such as high-resolution photography, three-dimensional modeling, and multimedia presentations to highlight key performance techniques, including pole manipulation and coordinated human–puppet interaction. Presenting these elements in visually accessible formats may help audiences better perceive the artistic value of puppetry, particularly in online and social media environments, where attention is highly fragmented. Second, since Flow Experience plays an important role in facilitating cultural identification, interactive and participatory programs should be further developed. For example, short-term workshops, hands-on demonstration sessions, or simple virtual reality applications may be introduced to allow visitors to directly experience basic puppetry techniques. By encouraging active involvement rather than passive observation, such programs may enhance emotional immersion and strengthen audience attachment to the cultural content. Third, in view of the dominant influence of Cultural Identity on Participation Intention, communication and outreach strategies should pay greater attention to conveying the social and historical significance of Liaoxi Puppet Opera. In addition to presenting artistic skills, exhibitions and promotional materials may include information about the endangered status of the art form, its regional origins, and its role in local collective memory. Framing public participation as a form of cultural responsibility rather than simple consumption may help foster a stronger sense of ownership and long-term commitment among audiences.

Implications for Younger Audiences. The young group accounts for a relatively high proportion of the sample in this study, which happens to provide a specific perspective for the contemporary activation of non-heritage. Unlike the older generation, today’s young people mainly experience traditional culture through digital media, rather than experiencing community activities in person. For this group, our data shows that visual appeal and immersive experience are important “grabs” to stimulate their initial interest. But more importantly, only under the drive of strong cultural identity can this brief online attention be truly transformed into a long-term protection behavior. This means that the digital communication strategy for young people must not be limited to the “good-looking” surface. To achieve true cultural inheritance, the digital expression of intangible cultural heritage must closely combine profound cultural connotations with novel visual forms, to guide the younger generation to consciously become the future guardians of endangered cultural heritage.

Overall, these implications suggest that effective revitalization of Liaoxi Puppet Opera requires a combination of enhanced visual presentation, increased opportunities for embodied participation, and value-oriented communication strategies. By aligning management practices with the psychological mechanisms identified in this study, practitioners may improve both short-term engagement and long-term support for heritage preservation.

## Conclusion

Based on the S-O-R framework, this study constructs and empirically verifies a structural model, which aims to clarify the psychological cognitive mechanism associated with the public to participate in the protection of puppetry in Liaoxi. The research results show that the unique visual aesthetics of puppetry, as a powerful environmental stimulus, effectively activates the organismic state that includes Flow Experience and Cultural Identity, thus significantly enhancing Participation Intention. Crucially, this study identifies the dual path mechanism of endangered heritage consumption and defines its different functional roles: the pleasure path (Flow Experience) acts as an attention hook and uses sensory immersion to make the audience detach from reality, while the realization path (Cultural Identity) acts as a behavioral anchor to awaken the audience’s deep sense of responsibility. Empirical evidence confirms that the latter is the dominant factor that fosters sustainable participation, and its influence far exceeds the mere pleasure.

In addition, the confirmation of the chain intermediary effect highlights a key portal mechanism, revealing that aesthetic immersion itself is not the end, but a psychological bridge that promotes the transition from sensory satisfaction to cultural resonance. Therefore, this study proposes that the revitalization of endangered ICH requires a paradigm shift from static object preservation to dynamic subject activation. Effective protection strategies must adopt a balanced approach: not only using digital aesthetics to induce Flow but also using cultural narrative to awaken Cultural Identity and finally cultivate a living transmission mechanism promoted by internal psychological forces.

## Limitations

Although this study has yielded valuable conclusions, certain limitations exist, which also provide directions for future research. First, this study employed a cross-sectional design, which can only reflect the psychological state of respondents at a specific point in time, making it difficult to capture the dynamic relationships between variables over time. Crucially, such cross-sectional data preclude the inference of causality; therefore, the identified sequential paths should be interpreted as theoretical associations rather than demonstrably causal mechanisms. Future research could consider adopting longitudinal methods, experimental designs, or time-lagged studies to rigorously verify causality and observe the formation process of Cultural Identity and its sustained impact on long-term protection behaviors. Furthermore, while the quantitative SEM approach employed in this study provides robust statistical validation for the proposed psychological pathways, it inherently lacks the nuanced, contextual depth offered by qualitative methodologies. To overcome the limitations of a single quantitative design, future research is strongly encouraged to incorporate anthropological observations and in-depth interviews. Such mixed-method approaches would help triangulate the current findings and capture the rich, lived experiences of audiences engaging with endangered ICH.

Second, regarding sample representativeness, the survey was distributed online using convenience and snowball sampling, resulting in a predominantly young (71.1% aged 18–35) and highly educated sample. As noted in our data collection, this predominance of younger respondents reflects our online sampling strategy directed at digitally engaged users, which inherently limits the generalizability of the findings to older populations. Consequently, the current findings primarily reflect the perceptions of younger demographics and cannot sufficiently represent evaluations across multiple, diverse age groups. Furthermore, the age classification used in the survey (e.g., grouping ages 35–60 into a single category) lacked rigorous granularity. Therefore, the results should be interpreted with caution and cannot be generalized to the broader public without further validation. This limits the extrapolation of our findings, as it may not fully reflect the perceptions of core local communities, older audiences, or less digitally engaged groups. Future research should employ stratified or random sampling to include a more demographically diverse population. Third, the study’s ecological validity and measurement of behavior have certain constraints. The respondents evaluated a curated package of one 2-min clip and eight still images, which may capture reactions to specific stimuli rather than the broader heritage form. Additionally, the study measured self-reported participation intention, which does not necessarily guarantee actual, sustained safeguarding behavior. Furthermore, relying primarily on self-reported questionnaires may be subject to subjective bias. Future research could integrate longitudinal behavioral tracking or neuroscience methods (such as EEG or eye-tracking) to more objectively measure viewers’ actual engagement and physiological arousal while watching Puppet Operas, thereby providing physiological evidence for the S-O-R mechanism. Finally, this study focused exclusively on the specific ICH project of “Liaoxi Puppet Opera, “and the generalizability of the findings remains to be further verified. Given the diverse types of ICH in China (e.g., embroidery, paper cutting, traditional opera), different mechanisms may exist for different categories. Future research could expand to other ICH categories or conduct cross-cultural comparative studies to test the boundary conditions of the proposed model.

## Data Availability

The original contributions presented in the study are included in the article/supplementary material, further inquiries can be directed to the corresponding author.
